# Physician Productivity and Supervision

**DOI:** 10.5811/westjem.60876

**Published:** 2023-05-09

**Authors:** Kraftin E. Schreyer, Diane Kuhn, Vicki Norton

**Affiliations:** * Lewis Katz School of Medicine at Temple University, Department of Emergency Medicine, Philadelphia, Pennsylvania; † Indiana University School of Medicine, Department of Emergency Medicine, Indianapolis, Indiana; ‡ Florida Atlantic University, Department of Emergency Medicine, Boca Raton, Florida

Emergency physician (EP) productivity has traditionally been measured in terms of patients per hour and has historically been estimated to be anywhere from 1.8 to 5.0, with most estimates ranging from 2.4 to 3.3.^1^ However, these early approximations from 20–40 years ago were derived from generalizations and individual conjecture. Furthermore, they largely failed to account for patient acuity, which has only risen since the inception of emergency medicine (EM) and even more so since the COVID-19 pandemic. Productivity has also come to be measured in other ways, which adds complexity to the original metric. The EM landscape today is very different than when those original values were proposed and, therefore, a fresh look at productivity is merited.

Productivity is closely tied to quality of care and patient safety. It is generally accepted that there is a trade-off between the number of patients evaluated per shift and the time and attention devoted to each of those patients. As more higher acuity patients are cared for during a shift, fewer overall patients can be evaluated; as more lower acuity patients are cared for during a shift, more overall patients can be evaluated. There is likely a threshold beyond which quality of care and safety are potentially sacrificed for efficiency and throughput. Determining that threshold, though, is very challenging, because EP and non-physician practitioner (NPP) productivity is influenced by a multitude of variables, many of which are constantly fluctuating. Because of the variability among these factors in all emergency departments (ED) and limited recent data, it is difficult, if not impossible, to identify a specific safe productivity threshold for EPs or NPPs.

In the following sections, we aim to outline the factors that affect productivity and supervision, and how those factors are likely to increase or decrease the number of patients that can be evaluated safely during a shift in the ED. We define productivity in terms of patients per hour evaluated during a shift in the ED. Primary productivity refers to the number of patients seen only by an attending EP. Overall productivity includes all patients evaluated during that shift, whether independently by an attending EP or by an attending EP in conjunction with a resident physician or NPP.

To supplement the existing literature with current data, we recently conducted a survey of practicing EPs who work in diverse clinical settings for a variety of employers. The relevant results are incorporated into the following discussion.

## PATIENT ACUITY

Productivity is routinely evaluated in the context of patient acuity. Higher acuity patients often require more complex thinking and decision-making, in addition to needing more resources for care. Higher acuity patients also often merit more documentation, which requires additional physician time.^1^,^2^ The additional time spent on each complex patient likely negatively impacts the overall efficiency of an attending EP. In a previous survey, academic EDs were found to have a higher rate of admission as compared to their community counterparts, suggesting that the patients are more complex. However, other markers of patient acuity, including the admission rate of patients arriving via emergency medical services and Current Procedural Terminology codes, were similar between academic and community settings, implying that the acuity mix is similar across different types of practice locations.^2^ Therefore, at either community or academic sites, we believe that greater numbers of higher acuity patients are associated with reduced primary and overall productivity.

While higher acuity patients generally require more treatment time, lower acuity patients can also merit additional clinician time beyond what their triage level may dictate. This may come in the form of answering questions the patient may have or reassuring patients about the absence of emergent diagnoses. Any additional time spent caring for lower acuity patients may also negatively impact productivity. However, while an increase in this patient subset would reduce primary productivity, it likely would have no impact on overall productivity.

Our survey found that the median number of patients per hour seen by practicing EPs, without supplementation from NPPs or resident physicians, was 2.1 patients per hour. This is lower than prior productivity estimates and is likely reflective of a patient acuity mix that now includes more higher acuity patients. However, of the respondents surveyed only two-thirds felt that they were able to see that many patients per hour in a safe manner.

## DOCUMENTATION

Documentation accounts for a significant portion of the time spent caring for individual patients in the ED, as it does in other clinical settings. Generally, reduced time documenting equates to more time available to see new patients, which would then lead to increased productivity.

The implementation of an electronic health record (EHR) has been shown to have mixed impacts on productivity, depending on the time the EHR has been in use. Early on, EHRs were shown to decrease productivity. Over time, however, productivity returned to baseline for the primary care practices that were studied.^3^ The same trajectory is likely true in EDs.

Scribes have been shown to both directly and indirectly increase physician productivity.^4^–^6^ By reducing the time required for the physician to directly document on each patient, physicians are able to see additional patients during each shift. A newer adjunct to documentation, voice recognition and dictation software, has been shown to reduce documentation time for nurses.^7^ Presumably, the same would hold true for physicians. Any documentation enhancement that shortens the time physicians must spend directly documenting will likely lead to an increase in both primary and overall productivity.

## EMERGENCY DEPARTMENT OPERATIONS

Department flow is maintained through three critical servers: beds; clinicians; and nursing. Boarding negatively impacts EP productivity. By definition, boarding patients occupy existing ED treatment spaces and reduce the capacity of that server. Occupied beds reduce the number of available beds for new patients. When new patients arrive but cannot be bedded in treatment areas, they instead occupy the waiting room. As this scenario has unfortunately become more common, physicians are seeing and evaluating patients in waiting rooms. This practice is not ideal, but it is necessary in many settings to allow patients to receive care. Physicians cannot see as many patients if they cannot be bedded; thus, both primary productivity and overall productivity are inherently reduced. Several survey respondents confirmed that flow in their EDs has been compromised by boarding, and as a result patient safety has been jeopardized.

In many EDs, EPs have responsibilities that go beyond their usual ED duties. These include responding to deteriorating patients or codes, staffing ED observation units, covering inpatient medical units, and accompanying ambulance transfers. The more duties a physician has beyond the ED, the less time there is to see and treat ED patients; thus, both primary productivity and overall productivity will decrease.

## STAFFING

Ancillary staff are critical to maintaining ED flow. Decreased nurse staffing is one factor that may decrease productivity. With fewer nurses, another of the three key servers for ED flow is compromised, which means that fewer patients can move through the department successfully. Furthermore, the remaining nurses may carry higher patient-nurse ratios, which requires them to divide their time and resources among more patients. Because of the server limitation, compounded by increased workload on the rest of the staff, EPs will not be able to see as many patients when there are nurse staffing shortages. In a nursing shortage, both primary and overall productivity would be reduced. Many survey respondents identified a shortage of nurse staffing as a barrier to providing safe patient care.

The same is true, to a lesser extent, for other ancillary staff such as patient care technicians and paramedics. While not one of the traditional ED critical servers for patient flow, non-nurse ancillary staff are adjuncts to expediting patient care and essential in many large-volume EDs. As is the case with nursing staff, the fewer additional ancillary staff who are available, the less time each patient can receive from those staff members. The less time a patient receives care from ancillary staff, the less is done to progress their care. Often that leads to a longer ED stay. Again, with shortages of non-nurse ancillary staff, both primary and overall productivity would be reduced.

## EXPERIENCE

The years of practice experience of all clinicians in a supervisory relationship is expected to impact clinical productivity. Generally, more practice experience should be associated with higher levels of clinical productivity. However, this is unlikely to be a linear relationship. Among attending EPs, we expect that clinical productivity increases over the first years in unsupervised practice as physicians form practice patterns and risk tolerance. There is likely a greater increase in primary productivity compared to overall productivity, as there is an additional learning curve for supervision.

Peak primary and overall productivity is likely to be reached when EPs are comfortable in the system in which they are working and have a set of safe heuristics that allows them to operate efficiently. However, this increase in clinical productivity is unlikely to continue over a career. Attending EPs in the late stages of their career may be less productive, both individually and overall, than they were in mid-career. This is likely the combination of discomfort with changing clinical practice conditions (eg, documentation changes), lower risk tolerance (as might occur after involvement in a lawsuit), and expected cognitive and physical changes with age.

For learners being supervised, more practice experience will likely correlate with increased autonomy and less supervision time needed to ensure clinical safety. Thus, a resident in their final year of training would require less supervision than an intern in the same program.

For NPPs being supervised, more practice experience in EM likely correlates with less supervision time and/or a lower level of supervision needed. The addition of NPPs has been shown to have mixed impacts on productivity. One study found that NPPs increase physician productivity, both in low- and high-acuity settings.^8^,^9^ Another found that NPPs increased productivity compared to resident trainees.^6^ However, a third study reported that when physicians were paired with NPPs, physician productivity decreased.^8^ Years of experience in EM likely impacted those results but were not fully accounted for. Independent of years of experience, however, EPs are more productive than NPPs. The Emergency Department Benchmarking Alliance typically assigns NPPs a lower productivity factor than EPs.^8^

A previous comprehensive survey found that attending physicians at community sites saw similar numbers of patients per hour, on average, with and without NPP coverage. However, when accounting for resident coverage at academic sites, attending EPs saw fewer patients per hour than their community counterparts.^2^ This implies that even though academic sites have residents that function as an extension of the attending EP, the supervisory requirement for trainees offsets the efficiency they may add. Resident supervision likely has more of a negative impact on efficiency because the supervisory requirements are more stringent vs the supervision of NPPs.^2^,^6^ In addition to EM residents, residents in other specialties are often intermediaries for consultations or admissions, which may further reduce efficiency. However, the higher level of supervision likely equates to a higher level of patient safety and lower rate of adverse events. The balance between efficiency and safety needs to be accounted for when comparing NPP and resident experience and supervision.

Our survey confirmed that more experienced NPPs increase overall physician productivity and that those NPPs with EM experience require less oversight than NPPs who have spent less time in EDs. While an increase in overall productivity would be expected with an increasing level of experience for both physician learners and NPPs, it is also likely that, with decreasing levels of experience, overall and primary productivity would be negatively impacted.

## SUPERVISION

Supervising the care provided by lesser trained clinicians (both learners and NPPs) is an integral part of both academic and community EM practice. In some practice settings, attending EPs do not see primary patients but rather devote their time to supervision of one or more clinicians.

The American Academy of Emergency Medicine (AAEM) believes that ED patients should have timely and unencumbered access to the most appropriate care led by a board-certified or board-eligible EP. The AAEM has made its position on supervision of NPPs by EPs clear in previous statements.^10^

Further, training of future EPs requires supervision and training of residents. The Accreditation Council for Graduate Medical Education (ACGME) has established that “[s]upervision in the setting of graduate medical education provides safe and effective care to patients; ensures each resident’s development of the skills, knowledge, and attitudes required to enter the unsupervised practice of medicine; and establishes a foundation for continued professional growth. . .Each patient must have an identifiable and appropriately credentialed and privileged attending physician. . . who is responsible and accountable for the patient’s care.” The ACGME further established that “[i]t is important that each program maintain sufficient levels of faculty staffing coverage in the Emergency Department in order to ensure adequate clinical instruction and supervision, as well as efficient, high quality clinical operations. The ACGME Review Committee uses a faculty staffing ratio of 4.0 patients per faculty hour or less as a guideline in this determination.”^11^,^12^

Overall, inadequate data is available regarding the impact of supervision and different models of supervision of residents and NPPs on EP productivity. Nonetheless, some basic principles may be expected to hold. First, the time and effort required to provide safe supervision decreases the number of patients that the attending EP can safely manage on their own (“primary patients”). Second, while the supervision of learners and the supervision of NPPs may contain overlapping features, the nature of these relationships is distinct. The clinical supervision of learners, both at the medical student and resident level, is a mentoring relationship in which the focus is on development of the learner into an independent EP. The relationship emphasizes both teaching and the provision of safe clinical care. In contrast, the clinical supervision of NPPs is centered around ensuring the provision of safe clinical care. Thus, the time and effort required for these distinct supervisory relationships is not comparable. More research in this area is an essential next step to inform policy.

The level of supervision needed in the supervisory relationship impacts attending EP productivity. Under direct supervision, which is the model expected for learners, attending EPs personally evaluate all patients. Under indirect supervision, attending EPs provide real-time guidance in patient evaluation and management but do not personally evaluate patients. In an indirect supervision model, attending EPs should have the ability to pivot to a direct supervisory role and evaluate patients if the need arises. Supervision should never be performed remotely. Remote supervision does not allow for the possibility of any direct supervision. Furthermore, we believe that an independently licensed and board-certified physician should be on site at all times in EDs and that remote supervision contradicts that tenet.

The decision regarding the level of supervision (eg, direct, indirect) required for any given situation should be made by the supervising EP and not by other stakeholders, including the individual being supervised or non-clinicians. While asynchronous chart review may serve as a quality assurance (QA) or human resources function, it does not represent a form of supervision, nor does it imply a physician-patient relationship between the physician reviewing the chart and the patient receiving care from another clinician. Furthermore, the asynchronous nature of the chart review suggests that it should not impact clinical productivity. More research is needed to determine appropriate compensation for the administrative and QA work associated with asynchronous chart review. If an EP is sent the chart of a patient whose clinical care they did not supervise and the EP does not have a compensated administrative review role, they should indicate this and, when appropriate, forward the chart to their administrative leadership.

Our survey of practicing EPs evaluated current practices and opinions on safety with respect to NPP supervision. The majority of our survey respondents who supervised NPPs oversaw two at a time, although a one-to-one ratio was preferred for direct supervision. The most common model of supervision was indirect supervision. Only two-thirds of survey respondents felt that their current supervision model was safe. Of the third who did not, about half recommended a direct supervision model to ensure safe care. A third of that group recommended additional training for EM-specific NPPs.

Regardless of the level of supervision, an increase in the number of clinicians that require supervision will reduce the primary productivity of a supervising attending EP. However, as those being supervised are able to see additional patients, overall productivity will likely increase. It should be expected, however, that the more supervision required, the more significant the reductions that will be seen in primary productivity, as well as in some reduction in overall productivity. Again, the balance between productivity and patient safety must be considered when evaluating supervision models.

## CONCLUSION

Physician productivity is impacted by several variables in a multitude of ways. While general trends can be identified, it is difficult to establish a direct numeric relationship between a change in the variables and the resultant impact on productivity. Our survey, with a median of 2.1 patients per hour, suggests that productivity is lower than prior estimates and is likely a combination of changing patient acuity, barriers to ED flow, and staffing limitations. Our findings further suggest that direct supervision is much safer than indirect supervision, and that the appropriate ratio for direct supervision is one EP to one NPP. While productivity can be enhanced by resident physicians and NPPs, maintaining a balance between productivity and safety must be a priority. Further exploration of the safety of supervision models and how those relate to productivity is merited. Changes to current supervision practices to optimize patient safety, while maintaining productivity, are necessary.
